# Artificial intelligence to evaluate the impact of urban green and blue spaces on chlorophyll-a concentrations

**DOI:** 10.1007/s11356-025-36292-9

**Published:** 2025-03-22

**Authors:** Panchali U. Fonseka, Lakindu Mampitiya, Namal Rathnayake, Hongsheng Zhang, Chaminda Samarasuriya, Ranjith Premasiri, Upaka Rathnayake

**Affiliations:** 1 Department of Earth Resource Engineering, Faculty of the Engineering, University of Moratuwa, Katubedda, Moratuwa, 10400 Sri Lanka; 2 Arthur C Clarke Institute for Modern Technologies, Katubedda, Moratuwa, 10400 Sri Lanka; 3 Water Resources Management and Soft Computing Research Laboratory, Athurugiriya, Millennium City, 10150 Sri Lanka; 4 Department of Civil Engineering, Faculty of Engineering, The University of Tokyo, 1 Chome-1-1 Yayoi, Bunkyo City, Tokyo, 113-8656 Japan; 5 Department of Geography, Centennial Campus, The University of Hong Kong, Pokfulam Road, Hong Kong, China; 6 Department of Civil Engineering and Construction, Faculty of Engineering and Design, Atlantic Technological University, Sligo, F91 YW50 Ireland

**Keywords:** Artificial intelligence, Chlorophyll-a concentrations, Random forest classification, Urban green and blue spaces, Urbanization

## Abstract

Urbanization is accelerating rapidly, highlighting the critical role of aligning with sustainable development goals, urban green and blue spaces (UGS and UBS). These spaces play a crucial role in enhancing the health and well-being of city residents in terms of ecology. Acknowledging the importance of urban ecology, this study utilizes Sentinel-2A data and support vector machine classification, aimed to identify UGS and UBS. To examine the connections between UGS and UBS, specific indices, spectral bands, and textures were calculated. Additionally, the concentration of chlorophyll, a vital indicator of ecological health, was assessed using three indices. Structural equation modeling was employed to elucidate the relationship between UGS and UBS and their impact on chlorophyll concentration for the years 2017 and 2023. In the 2017 model, UGS exhibited a positive path coefficient (0.25) with chlorophyll-a, indicating that an increase in UGS is associated with an increase in chlorophyll levels. Conversely, in 2023, the path coefficient turned negative (− 0.83), presenting a stark contrast to the 2017 model. This shift suggests potential environmental or urban development changes, such as alterations in the quality or type of urban green spaces, potentially including more non-native or ornamental plants that contribute less to overall chlorophyll levels. UGS can be subjected to pollution, soil compaction, and other stressors that reduce plant health. Similarly, the UBS showed an increase in its path coefficient from − 0.99 in 2017 to − 1.8 in 2023, suggesting improvements such as cleaner water or urban planning strategies aimed at reducing water pollution. The consistent negative relationship across both years suggests that urban water bodies are not contributing to Chl levels due to complex interactions of water bodies with their urban surroundings. However, further research is essential to delve into these dynamics and comprehend the implications for urban ecological planning and sustainability.

## Introduction

The continuous expansion of cities has progressively transformed urban ecological landscapes, creating significant spatial variations that exacerbate global warming and intensify urban heat islands (UHIs) (Liu et al. 2024). In this context, urban green space (UGS) and urban blue space (UBS) are pivotal in adaptable urban thermal environments (Shi et al. 2020). These elements not only play a critical role in regulating urban temperatures but also in creating more livable urban spaces through processes like shadowing, evapotranspiration, and air movement modulation (Shi et al. 2020; Wu et al. 2019). UBS, through its inherent properties, aids in temperature regulation by facilitating energy exchange with water bodies, thus offering a cooling effect similar to that of UGS during warm conditions. Furthermore, UGS, such as parks, gardens, and street trees, along with UBS, including rivers, lakes, and ponds within city boundaries, enhance urban biodiversity, improve air quality, and regulate microclimates (Kumar et al. 2023). The efficiency of these spaces in temperature moderation is influenced by various factors including their design, size, type, and the urban layout surrounding them (Pritipadmaja et al. 2023; Wang et al. 2023).

Similar to UGS and UBS, in the urban environment, chlorophyll plays a crucial role. Chlorophyll concentration serves as an indicator of plant health and productivity, reflecting the capacity of urban vegetation to perform photosynthesis under varying urban conditions (Bramich et al. 2021; Zarco-Tejada et al. 2019). In UBS, the Chl-a concentration acts as a reliable proxy for assessing the health and quality of water bodies, serving as a robust indicator of their trophic status (Buma and Lee 2020). Chl-a, derived primarily from phytoplankton, is widely used to infer the ecological state of lakes and the clarity of water bodies (Ayeni and Adesalu 2018; Barraza-Moraga et al. 2022; Sòria-Perpinyà et al. 2019). Phytoplankton, as a primary component of Chl-a, plays a key role in determining the trophic state of aquatic systems (Barraza-Moraga et al. 2022). Chlorophyll concentration is typically measured using various laboratory and field methods, including spectrophotometry, fluorometry, and high-performance liquid chromatography (HPLC) (Ayeni and Adesalu 2018; Barraza-Moraga et al. 2022; Sòria-Perpinyà et al. 2019). While these methods provide accurate quantification by extracting chlorophyll from samples, they are often limited in their ability to assess large areas comprehensively (Ouma et al. 2020; Sent et al. 2021).

The advancement of modern technological tools like geographic information systems (GIS) and remote sensing (RS) have revolutionized environmental monitoring and management. These tools enable the coverage of large areas and facilitate the acquisition of data from inaccessible locations, surpassing the limitations of traditional studies (Barraza-Moraga et al. 2022; Ouma et al. 2020). They have become essential in monitoring and managing environmental changes effectively, utilizing multi-temporal satellite imagery for quantitative assessments (Barraza-Moraga et al. 2022; Majumder et al. 2023; Rejaur Rahman and Rahman 2023). Remote sensing has been widely applied to identify UBS and UGS spaces, while its potential for monitoring chlorophyll-a (Chl-a) has expanded beyond the constraints of traditional active techniques (Ayeni and Adesalu 2018; Barraza-Moraga et al. 2022; Bramich et al. 2021; Zarco-Tejada et al. 2019). However, although GIS and RS provide valuable data on UBS, UGS, and chlorophyll, their interrelationships and combined impacts remain underexplored. Previous studies (e.g., (Wang et al. 2023; Wu et al. 2019)) have demonstrated the cooling effects of UGS and UBS but have not integrated Chl-a as a metric for their ecological health. The integration of advanced mathematical models and machine learning algorithms within GIS platforms has significantly enhanced the accuracy of detecting and monitoring this kind of environmental phenomena (Sharma et al. 2024; Wiatkowska et al. 2021).

Measuring chlorophyll-a in urban areas, particularly within UGS and UBS, is essential for developing effective climate mitigation strategies (Wu et al. 2019). This measurement provides insights into the health and efficiency of these spaces in performing crucial ecological functions, such as carbon sequestration, air purification, and temperature regulation. However, the relationship between UGS, UBS, and Chl-a is not yet well established. Research on the impact of UGS and UBS on Chl-a remains scarce, underscoring the need for comprehensive studies to understand and mitigate urban growth impacts on these critical ecological parameters. This research gap is particularly evident in tropical regions like Sri Lanka, especially in the Colombo Metropolitan Area (CMA), the most urbanized region and the only metropolitan area in the country (Khaniya et al. 2021). With CMA facing significant climate vulnerabilities, particularly concerning environmental degradation and the urban heat island (UHI) effect, regulating these vulnerabilities is crucial. This study addresses this critical gap by focusing on the interrelationships between UGS, UBS, and Chl-a in the CMA using structural equation model (SEM). It is the first of its kind in Sri Lanka, marking a pivotal contribution to ecological research and urban planning disciplines. By exploring these unexplored dynamics, this research offers valuable insights into sustainable urban development and environmental management in tropical urban settings.

## Materials and methods

### Study area

**Fig. 1:**
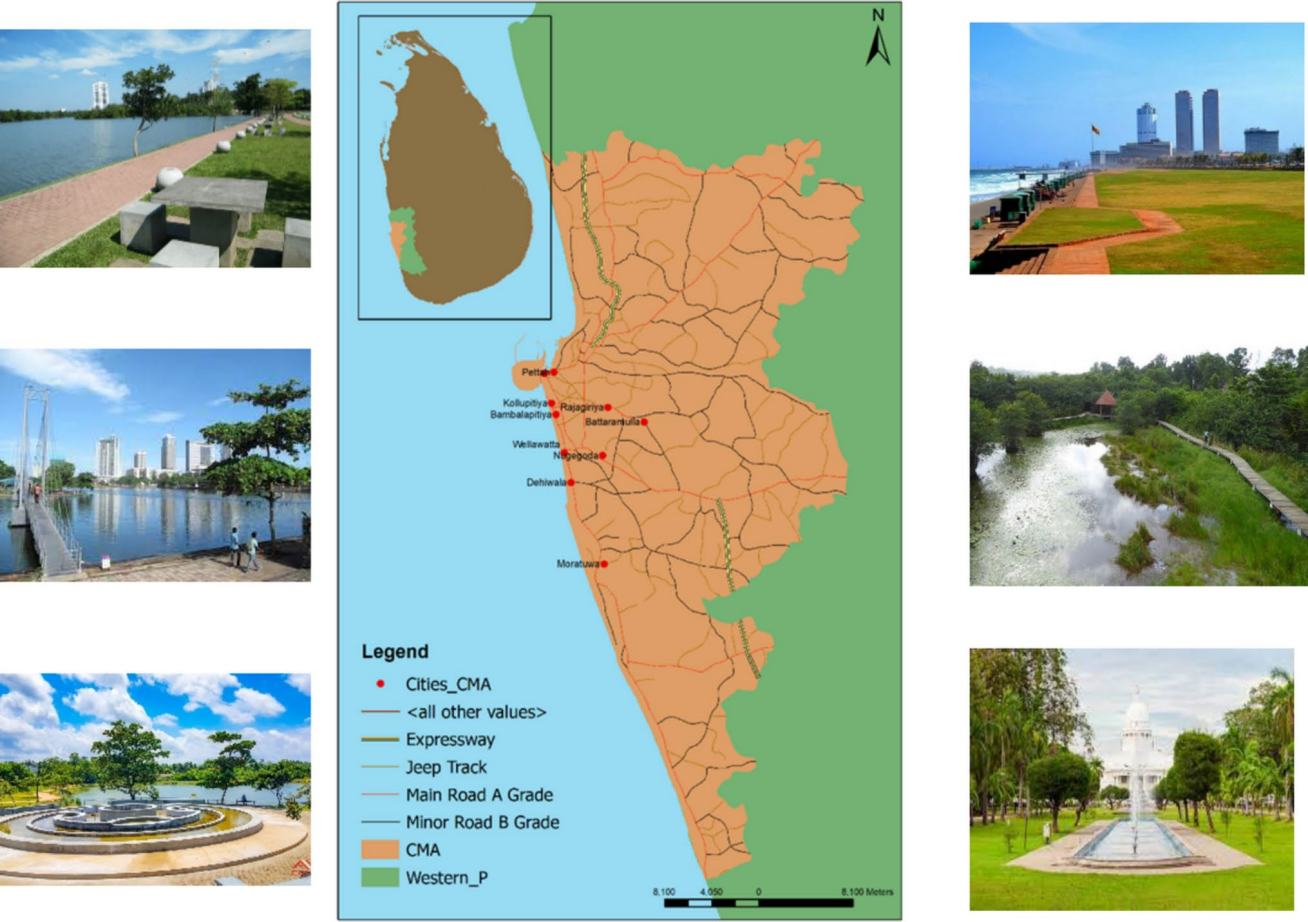
Colombo metropolitan area. The left column highlights examples of blue spaces, and the right column presents examples of green spaces

The Colombo Metropolitan Area is depicted in Fig. 1, chosen as the focal point for this study, as it stands out within the country as the only metropolitan and is also characterized by its high level of urbanization. Therefore, the study area became a critical area for urban ecological research. In the context of urban ecology, Colombo presents a compelling case study due to the significant ecological transitions that accompany its urban development.

By focusing on Colombo, the research aims to shed light on the interactions between urbanization and ecological factors, offering insights that are vital for sustainable urban development and environmental stewardship within metropolitan settings.

### Data collection

Identifying UGS and UBS within densely urbanized areas is a critical task. Satellite imagery has more advantages for its cost-effectiveness and ability to provide consistent data (Barraza-Moraga et al. 2022; Ouma et al. 2020; Sent et al. 2021). This study used Sentinel-2A multi-spectral images, chosen for their superior spatial and spectral resolutions compared to other satellites like MODIS or Landsat (Li et al. 2021). The images, corrected for atmospheric distortions to ensure accurate surface reflectance, span the years 2017 and 2023 to track the development of UGS and UBS over time.

To address seasonal variations and environmental factors like cloud coverage or terrain, we analyzed a comprehensive set of 64 features as detailed in Table 1 (Jeong and Park 2023). Employing a multi-temporal approach minimized data redundancy and noise, and composite images were created covering the first inter-monsoon season, southwest monsoon seasons, second inter-monsoon season, and northeast monsoon seasons, streamlining the analysis process (Hościło and Lewandowska 2019; Phan et al. 2020; Shetty et al. 2021).

**Table 1:** Band math for indices used in SVM classification

Category	Features	Explanation	Reference
Spectral bands	Band 2 Band 3 Band 4 Band 5/6/7 Band 8a Bands 11/12	Blue visible Green visible Red visible Red edge NIR SWIR	(Wessel et al. 2018); (Abdi 2020)
Spectral indexes	NDVI	$\frac{\mathrm{NIR}-\mathrm{RED}}{\mathrm{NIR}+\mathrm{RED}}$	(Goldblatt et al. 2016; Zhou et al. 2019)
	SAVI	$\frac{\mathrm{NIR}-\mathrm{RED}}{\mathrm{NIR}+\mathrm{RED}+\mathrm{L}}\times (1+\mathrm{L})$	
	EVI	$2.5 \frac{\mathrm{NIR}-\mathrm{RED}}{\mathrm{NIR}+6\times \mathrm{RED}-7.5\times \mathrm{Blue}+1}$	
	NDWI	$\frac{\mathrm{Green}-\mathrm{NIR}}{\mathrm{Green}+\mathrm{NIR}}$	
	NDBI	$\frac{\mathrm{SWIR}-\mathrm{NIR}}{\mathrm{SWIR}+\mathrm{NIR}}$	
	GCC	$\frac{\mathrm{Green}}{\mathrm{Blue}+\mathrm{green}-\mathrm{red}}$	
Texture	Contrast	$\mathrm{CON}=\sum_{\mathrm{i},\mathrm{j}=0}^{\mathrm{N}-1}{P}_{\mathrm{i},\mathrm{j}}{\left(i-j\right)}^{2}$	(Zhou et al. 2019)

### Methodology

**Fig. 2:**
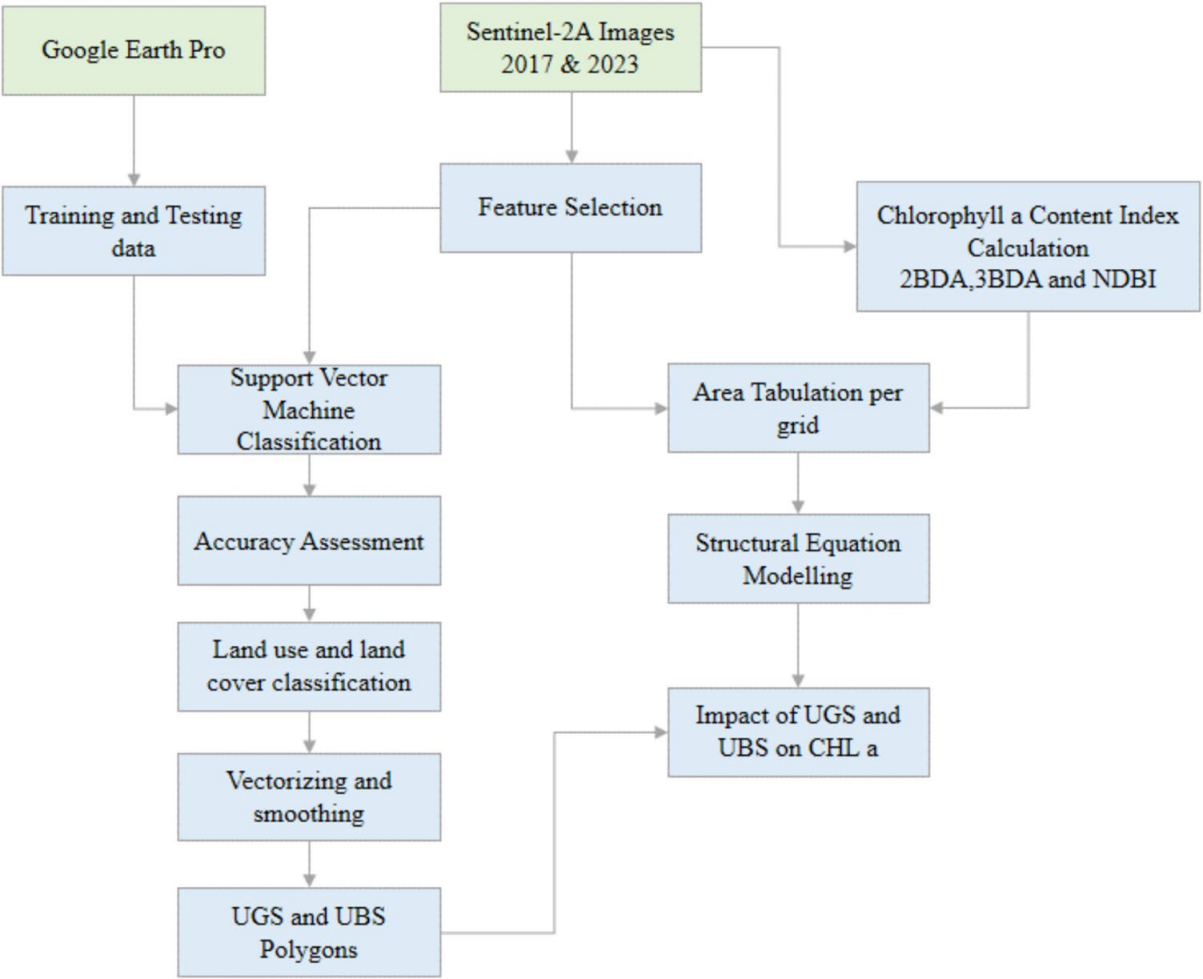
The working flow chart of this study

The complete methodology is summarized as depicted in Fig. 2.

#### Creating training dataset

**Fig. 3:**
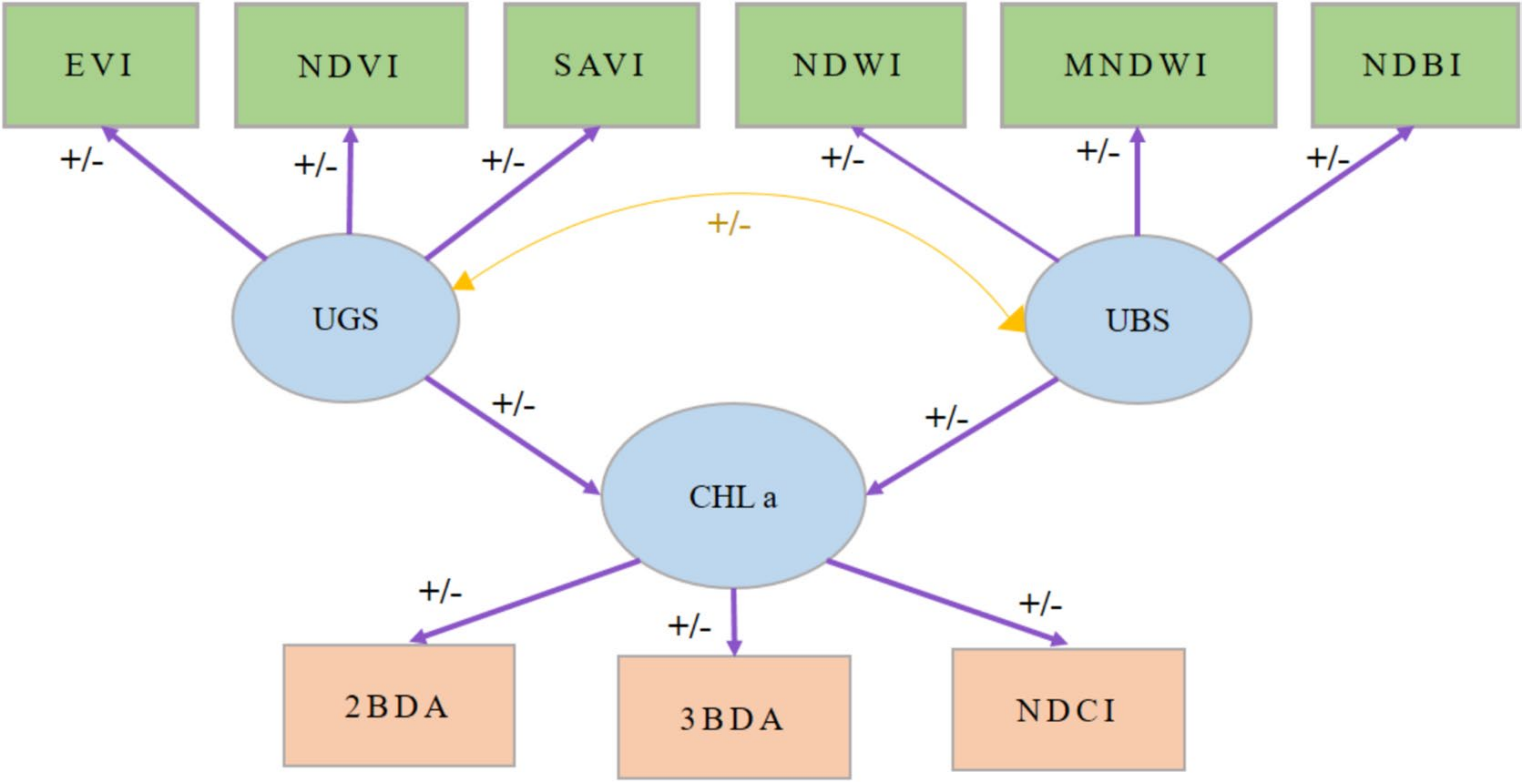
Foundational hypothesis of SEM results

In this research, the assembly of training datasets prepared with categorized data for machine learning classification appeared as a key phase. The challenge lies in accurately labeling UBS and UGS, with the labeling quality significantly impacting the effectiveness of the classification algorithm (Jeong and Park 2023; Shetty et al. 2021). To address this, seven distinct categories of land cover were defined: forests, grasslands, wetlands, bare land, croplands, urban areas, and water, as illustrated in Fig. 3. Sample points for these categories were collected using Google Earth Pro. Stratified random sampling was implemented for each land cover category to ensure a balanced representation (Jin et al. 2014).

To mitigate subjectivity and ensure consistency in labeling, a quality control process was applied. Labels were reviewed by experts with domain knowledge in land cover classification to validate the accuracy of the sample points (Moraes et al. 2024). To avoid bias in the training data, spatial autocorrelation (Arthur 2024) was addressed by maintaining a minimum separation distance between sampled points. A spatial thinning algorithm (Aiello‐Lammens et al. 2015) was applied to remove closely clustered points, ensuring that the dataset was unbiased and representative of diverse spatial conditions.

#### Support vector machine land cover classification

In this section, machine learning is employed for the classification of images in order to identify the UGS and UBS. Therefore, the extracted features of the sentinel images served as inputs for the SVM model (Arfa and Minaei 2024*)*. In the SVM classification, the principle of identifying the narrowest margin, or hyperplane, which best divides the data classes is employed (Thiyagarajan and Vijayalakshmi 2024*)*. Depending upon the dataset, the SVM algorithm uses an *N*-dimensional (*N*—number of features of the dataset) space (Ke et al. 2024; Thiyagarajan and Vijayalakshmi 2024) to identify the hyperplane that lies in between the classes. In the process of training of SVM model, the extracted dataset (forests, grasslands, wetlands, bare land, croplands, urban areas, and water) were cleaned and fed to the model. The preprocessing of Sentinel-2 imagery was performed using Google Earth Engine (GEE) (Teijido-Murias et al. 2024). Atmospheric distortions were removed using the Sentinel-2 Surface Reflectance product, processed with the Sen2Cor algorithm (European Space Agency 2025). Radiometric adjustments were inherently applied within the Surface Reflectance product to ensure consistency in pixel values across images. Cloud and shadow pixels were masked using the “QA60” (European Space Agency 2025) band, eliminating artifacts in the input data. The cleaned and preprocessed imagery was then used to derive spectral indices and texture features, as outlined in Table 1, to enhance the model’s ability to differentiate between land cover types. Model overfitting and undercutting were mitigated through the proper handling of the input data and the tuning of the hyperparameters of the model (Mampitiya et al. 2023). To optimize the performance of land cover classification maps, the study undertook hyperparameter tuning performance using a grid search approach (Mampitiya et al. 2023). Hyperparameter tuning is one of the crucial steps of machine learning. *γ* (kernel coefficient) and *c* (regularization parameter) hyperparameters were turned with respect to the functionality of the model. Among the different kernel types (polynomial, radial basis function, linear and Sigmoid kernel) (Keerthi and Lin 2003*)*, the radial basis function (RBF) was able to function over the dataset accurately due to its ability to handle non-linear boundaries effectively. The fine-tuned hyperparameters guided the generation of accurate urban land cover maps (ULCMs) for the study area. The model’s performance was evaluation evaluated of these models hinged on several key metrics: Overall accuracy (OA), F1-score reflecting both producer accuracy (PA) and user accuracy (UA), and Kappa index, which collectively provided a comprehensive measure of each classifier’s performance.

With the view from the evaluation matrices mentioned about, the performance of the model was validated. According to the result, the model was able to build a hyperplane to carry out the multi-class classification.

#### Estimating Chl-a concentration

This study incorporates four distinguished algorithms for the assessment of Chl concentration from satellite reflectance data: the two-band algorithm (2BDA), the three-band algorithm (3BDA), and the normalized difference chlorophyll index (NDCI). To align the resolution of Sentinel-2 (S2) imagery for algorithm application, band resampling was executed, particularly for 2BDA, 3BDA, and NDCI indices (Buma and Lee 2020), ensuring bands 4 (10-m resolution) matched the resolution of bands 5 and 8b (20 m) through nearest neighbor resampling (refer to Table 2). This method, chosen for its simplicity and data integrity preservation, resulted in 20-m resolution indices for 2BDA, 3BDA, and NDCI. The deployment of Sentinel-2 imagery has been demonstrated to achieve satisfactory accuracy in determining Chl-a levels.

**Table 2:** Band math for the algorithm used in Chl-a concentration

Category	Features	Explanation	Reference
Spectral bands	2BDA	$\left(\frac{\text{Band }5}{\text{Band }4}\right)$	(WATANABE et al. 2018)
	3BDA	$(\frac{1}{\text{Band }4}-\left(\frac{1}{\text{Band }5}\right)\times \mathrm{Band}8\mathrm{b}$	(Abdelmalik 2018)
	NDCI	$\left(\frac{\text{Band }5-\text{Band }4}{\text{Band }5+\text{ Band }4}\right)$	(Shahzad et al. 2018)

#### Relationship between UBS and UGS and Chl-a using SEM modeling approach

The influence of UBS and UGS on local Chl-a concentrations is analyzed using a structural equation model (SEM) (Fan et al. 2016). SEMs are practiced at testing causal hypotheses with empirical data, offering unstandardized interaction values similar to partial regression coefficients for direct interpretation, alongside standardized coefficients that highlight a predictor’s unique contribution to explaining variance in a response variable (Grace and Bollen 2008). One of SEM’s strengths is its ability to address multi-collinearity issues, positioning it as a superior alternative to traditional multi-variate regression in certain contexts (Hair et al. 2020; Jacobucci et al. 2019).

In this study, Chl-a levels, derived from Sentinel-2A data, serve as the endogenous variable, with UBS and UGS acting as exogenous variables (as depicted in Fig. 2). However, the variables in the rectangles are observed variables which are directly measured, and circles represent latent variables which are not directly measured but inferred from other observed variables. Figure 2 introduces the foundational hypothesis of our SEM analysis. Utilizing a point grid sampling method (illustrated in Fig. 1) yielded a sample size of 197 m^2^.

## Results and discussion

### Building training datasets with labeled data and features

**Fig. 4:**
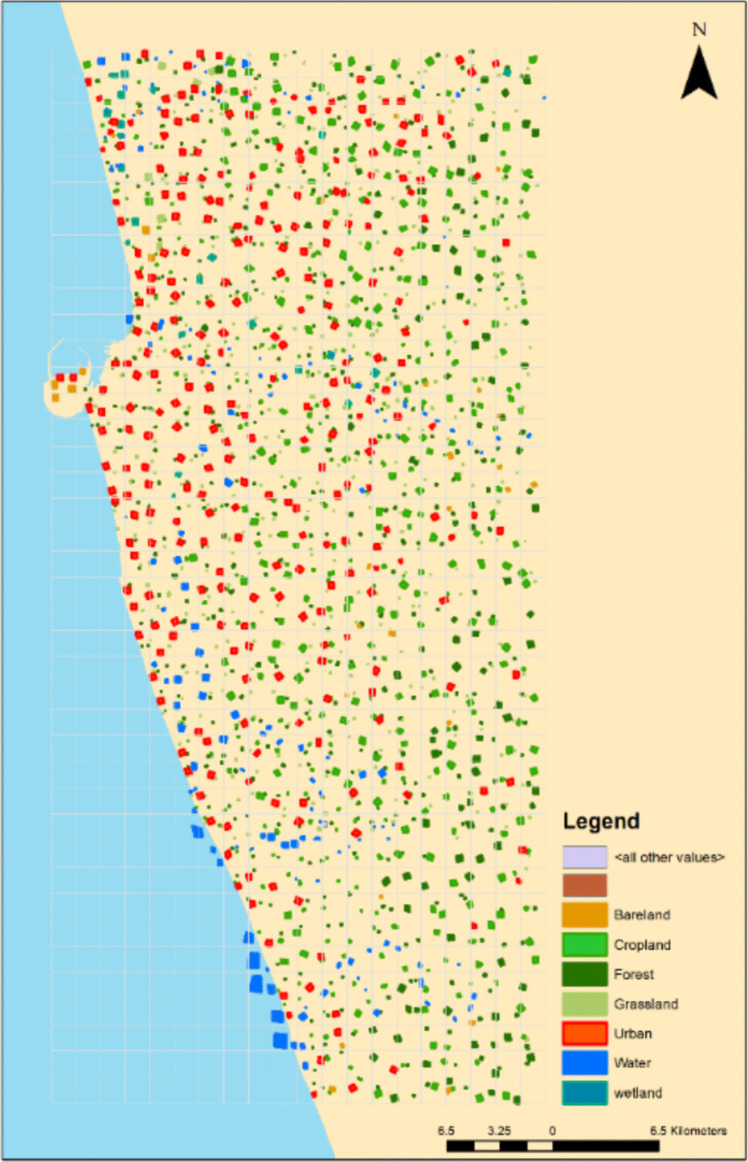
Distribution of training data set

By applying machine learning models to Sentinel-2A images, land use and land cover classes were created by using labeling data, as shown in Fig. 4. Although creating training data is expensive and time-consuming, it has an enormous influence on how well machine learning models. It is well known that the model’s classification accuracy increases with sample size (Moraes et al. 2024; Jin et al. 2014). When conducting research over a vast area, it will be important to compare the accuracy by sample size across different places. The less variable the SD of the model’s performance is when stability is taken into account, the more usable the model is.

### Land cover classification based on machine learning models

The study’s SVM classifier yielded an overall accuracy (OA) of 79.45%, a Kappa coefficient of 0.76, and an F1-score of 0.81 for the year 2017, indicating that the suggested mapping process effectively classifies land use in metropolitan settings. For 2023, the SVM classifier demonstrated an improved OA of 82.67%, a Kappa coefficient of 0.80, and an F1-score of 0.84, further validating the robustness of the classification approach. Due to their complicated designs and diverse land-use patterns, densely populated cities like the study region frequently have different land uses mixed together, sometimes even inside a single pixel. Due to their small size and intricate shapes, UGS and UBS are less accurately classified (Yan et al. 2023). Relatively well, the SVM model represented the connected form of the UBS and UGS.

**Fig. 5:**
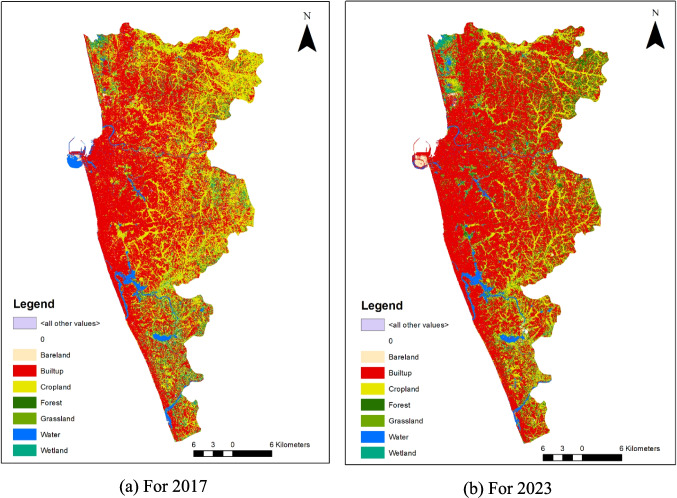
Land use and land cover analysis

The distribution of land cover highlights the significant characteristics of the study areas, as illustrated in Fig. 5. Visually, Fig. 5a and b display a high degree of similarity, primarily due to the urban expansion of the study area. Over the past decades, Colombo has undergone significant urbanization, characterized by infill urbanization which is known as densification of existing urban areas rather than expansion into surrounding non-urban land. A notable change is the development in the port city at the northwestern edge of the study area. Quantitatively, urban coverage increased from 52.6% in 2017 to 58.4% in 2023. However, the detection of urban green areas, forests, croplands, and grasslands was limited by the 10-m spatial resolution of the satellite images. Despite this, support vector machine (SVM) analysis proved to be excellent for visual interpretation, clearly identifying urban forests with minimal salt-and-pepper effect and enhanced connectivity (Rimal et al. 2020). During the study period, urban green spaces (UGS) decreased from 37.9 to 33.4%, while urban built-up spaces (UBS) increased from 3.0 to 3.23%. However, Sentinel images were challenged by noise and redundant information (Wessel et al. 2018). Reducing dimensions through principal component analysis or employing an optimal model that accurately reflects key variables, tested through various combinations, is essential.

### Estimating Chl-a concentration

Table 3 shows that from 2017 to 2023, the values for 2BDA, 3BDA, and NDCI decreased in bare lands and urban areas. This decrease mainly happened because the land changed due to the spread of cities. Urbanization or building activities changed the land’s surface, leading to less healthy plants. In wetlands, croplands, and forests, the index values slightly dropped, likely due to human activities (Assennato et al. 2022). However, grasslands showed a significant change in all indexes, indicating poor vegetation health. In the water category, 2BDA values stayed the same over the years, while 3BDA values went up, and NDCI values went down. This happened because different indexes react to different aspects of the water, especially the plants living in it, like aquatic vegetation (Assennato et al. 2022). The steady 2BDA values suggest it does not react much to changes in water. Overall, the table indicates that land use changes and human activities have caused environmental and ecological changes.

**Table 3:** Changes through 2BDA, 3BDA, and NDCI based on land cover classes from 2017 to 2023

	2BDA		3BDA		NDCI	
Class	2017	2023	2017	2023	2017	2023
Bare land	1.058	1.01	1.22	1.162	0.169	0.08
Wetland	1.142	1.074	1.36	1.163	0.278	0.17
Urban	1.065	1.028	1.39	1.152	0.216	0.11
Grassland	1.136	0.063	1.44	1.174	0.276	0.16
Forest	1.134	1.054	1.38	1.134	0.303	0.16
Cropland	1.136	1.054	1.36	1.117	0.285	0.15
Water	1.004	1.006	0.877	0.938	0.078	0.05

### Relationship between UBS and UGS and Chl-a using SEM modeling approach

**Fig. 6:**
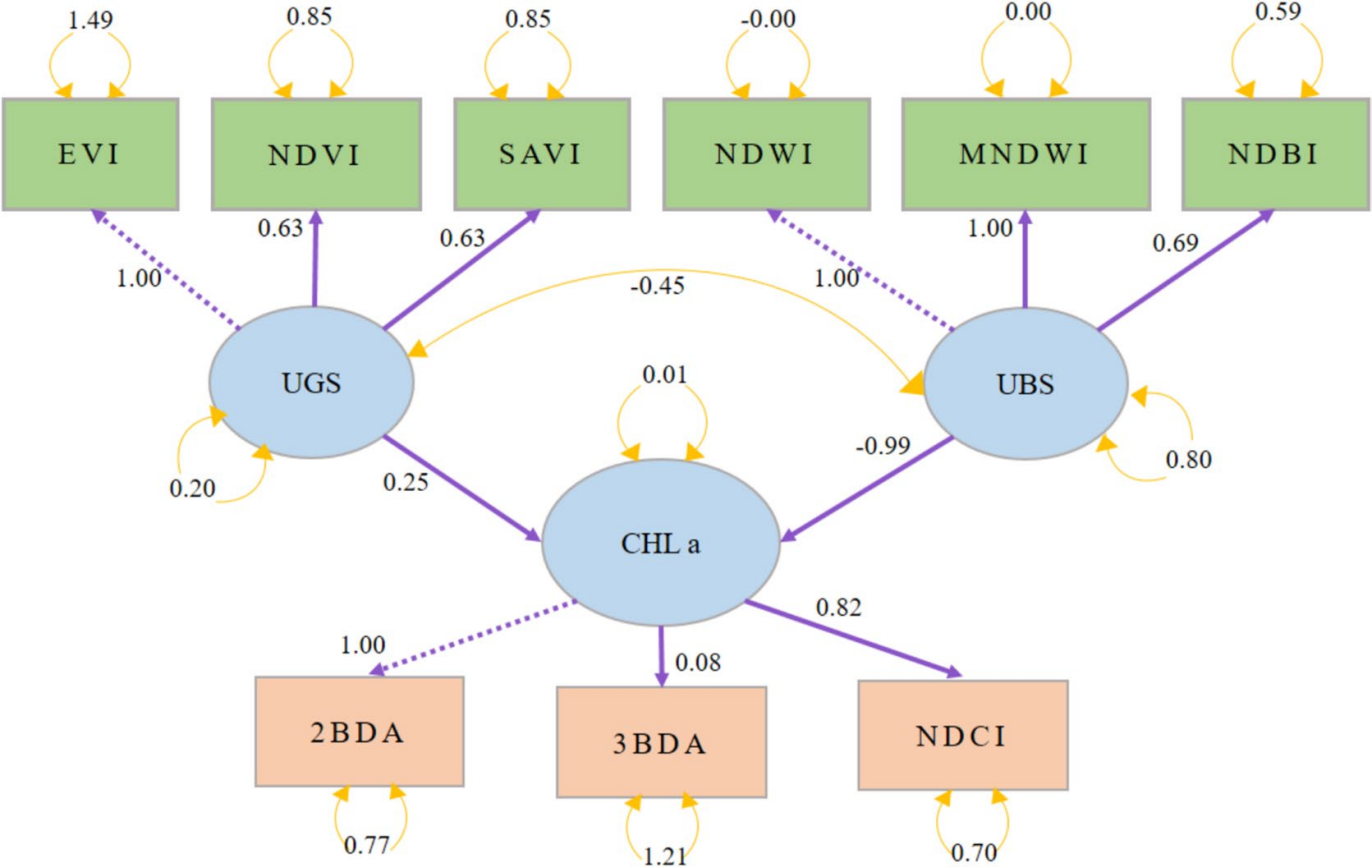
SEM for 2017

The relationship between UBS and UGS with Chl was analyzed using the SEM for 2017 and 2023 separately. The results are shown in Fig. 6, and the interactions among variables were well established by SEM. Goodness-of-fit (GOF) (Grace and Bollen 2008) measures of the final SEM were as follows: for 2017, comparative fit index (CFI) = 0.912, root mean square error of approximation (RMSEA) = 0.096, and standardized root mean square residual (SRMR) = 0.067; and for 2023, CFI = 0.892, RMSEA = 0.091, and SRMR = 0.058, indicating a good fit of the model.

In the 2017 model, UGS has a positive path coefficient (0.25) with chlorophyll (Chl), which suggests that an increase in UGS is associated with an increase in chlorophyll levels. On the other hand, UBS has a strong negative path coefficient (− 0.99) with chlorophyll, indicating that an increase in UBS is associated with a significant decrease in chlorophyll levels. This could suggest that in 2017, urban greenery positively contributed to the photosynthetic activity, reflected in higher chlorophyll levels (Zarco-Tejada et al. 2019). However, urban blue spaces seemed to have a detrimental effect. One possible explanation could be that urban blue spaces may not directly support vegetation in the same way green spaces do, or they could be indicative of urban development that reduces green cover (Ayeni and Adesalu 2018; Sent et al. 2021).

**Fig. 7:**
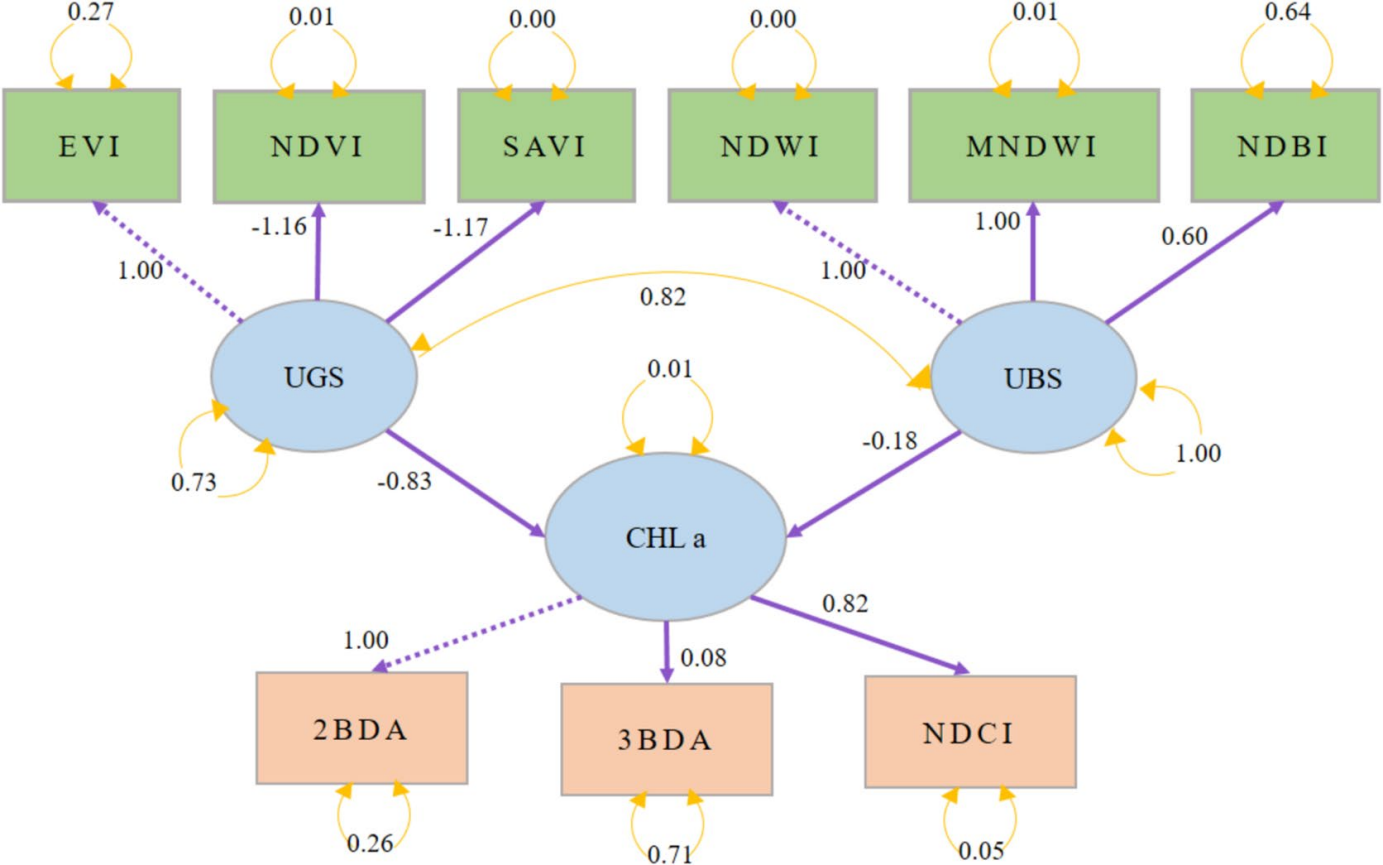
SEM for 2023

On the other hand, in the 2023 model (refer to Fig. 7), the relationship between UGS and Chlorophyll is negative (− 0.83), which is a stark contrast to the 2017 model. This suggests that, over time, the relationship between green spaces and chlorophyll levels has changed significantly, with an increase in UGS now associated with a decrease in chlorophyll. For UBS, the path coefficient in 2023 is also negative (− 0.18), which, while less negative than in 2017, still suggests that an increase in UBS correlates with a decrease in chlorophyll levels, albeit to a lesser degree.

The shift in the relationship between UGS and chlorophyll levels between 2017 and 2023 could indicate several possible environmental or urban development changes. For example, the quality or type of urban green spaces may have changed, potentially including more non-native or decorative plants that contribute less to overall chlorophyll levels (Tartaglia and Aronson 2024). UGS may be subjected to pollution, soil compaction, and other stressors that reduce plant health and chlorophyll production (Le Saint et al. 2024). Alternatively, the increase in UGS may be in areas that do not contribute effectively to the overall ecosystem's photosynthetic activity (Priya and Senthil 2024).

As for UBS, the consistent negative relationship across both years suggests that urban water bodies are not contributing to chlorophyll levels, which may not be surprising given that these spaces are typically less vegetated. While there is limited direct research on this specific relationship, a study focusing on Kobe, Japan (Zhang et al. 2024), evaluated the combined effects of blue–green spaces on the urban thermal environment. This study implies that while blue spaces may influence urban temperatures, their direct impact on chlorophyll levels, typically associated with vegetated green spaces is minimal. Additionally, Ampatzidis and Kershaw (2020) have highlighted the complex interactions of water bodies with their urban surroundings, showing varying effects on urban temperatures. However, the reduced negative impact over time might reflect better management practices or urban planning integrating blue and green spaces. For example, initiatives like the Metro Colombo Urban Development Project (Ministry of Urban Development and Housing 2025), which developed canal diversions and walking parks, may have contributed to this trend. Furthermore, the less negative relationship in 2023 compared to 2017 could indicate that urban blue spaces are being developed in a more environmentally sensitive manner, or that they are being better integrated with green spaces to support urban biodiversity.

While these findings are insightful, the study has several limitations that should be addressed in future research. The study does not explicitly account for intra-annual (seasonal) variability in land cover changes, which may influence the accuracy of classification and the relationship between UGS, UBS, and Chl-a concentrations. Additionally, this study focuses solely on SVM for classification, without comparing its performance to other machine learning algorithms such as random forests, decision trees, or CNNs. The results are specific to the CMA and the temporal windows analyzed (e.g., 2017 and 2023), which limits the generalizability of the findings to other regions or time periods. Furthermore, the lack of field-based validation or laboratory analyses for Chl-a quantification means that the accuracy of satellite-derived indices and classifications cannot be fully verified. Future studies should address these limitations by incorporating multi-seasonal and multi-regional datasets, comparing different machine learning approaches, and integrating ground-truth data to enhance the reliability and generalizability of the findings.

## Conclusions

The complex dynamics of urban expansion in developing countries present significant challenges to sustainable development, particularly in the integration and maintenance of UGS and UBS. These challenges are crucial because UGS and UBS contribute significantly to urban ecosystems by maintaining chlorophyll-a (Chl-a) levels, indicative of healthy photosynthesis and vegetation vitality (Sunita et al. 2023). These spaces are not only aesthetically and recreationally important but also serve key ecological functions, including carbon sequestration and air quality improvement (Wolch et al. 2014).

A notable shift in the structural equation modeling (SEM) study shows a positive relationship between UGS/UBS and Chl-a in 2017, indicating that urban greenery effectively supported plant health. In contrast, the 2023 data reveal a negative relationship, suggesting that recent urban planning practices or ecological changes may be compromising the ability of UGS and UBS to sustain plant life. This could result from the encroachment of urban development on green spaces, pollution increase, or the introduction of less beneficial non-native plant species.

Such findings resonate with the urgency highlighted in the IPCC’s Special Report on Global Warming (IPCC 2019b) of 1.5 °C, which calls for transformative changes in urban infrastructure and practices to mitigate climate change. The IPCC’s AR6 (IPCC 2019a) further emphasizes the role of green and blue infrastructure in urban cooling and climate adaptation. Similarly, the IPBES (2019) underscores the importance of biodiversity in urban spaces, which can be supported by robust UGS and UBS. These reports advocate for the protection of urban ecosystems, aligning with the goals of the Paris Agreement to limit global warming through nature-based solutions like maintaining healthy Chl-a levels.

The negative trends observed in 2023 underline a critical need for urban planning strategies that are adaptive and responsive to environmental changes. Prioritizing the conservation and enhancement of UGS and UBS can significantly contribute to urban ecological health and align with global sustainability goals, supporting the broader aims of the IPCC, the IPBES, and the Paris Agreement. In conclusion, this research suggests that maintaining and expanding urban greenery is not only beneficial for local ecosystems but is also integral to global climate change mitigation efforts. It calls for a strategic approach in urban planning to ensure that the development and management of UGS and UBS are factored into broader policy frameworks, thereby ensuring their continuous provision of ecosystem services and support of biodiversity in rapidly urbanizing landscapes.

## Data Availability

Data used in this manuscript can be requested from the corresponding author.
